# Tumor Margin Contains Prognostic Information: Radiomic Margin Characteristics Analysis in Lung Adenocarcinoma Patients

**DOI:** 10.3390/cancers13071676

**Published:** 2021-04-02

**Authors:** Geewon Lee, Hyunjin Park, Ho Yun Lee, Joong Hyun Ahn, Insuk Sohn, Seung-Hak Lee, Jhingook Kim

**Affiliations:** 1 Department of Radiology and Center for Imaging Science, Samsung Medical Center, Sungkyunkwan University School of Medicine, Seoul 06351, Korea; rabkingdom@naver.com; 2 Department of Radiology and Medical Research Institute, Pusan National University Hospital, Pusan National University School of Medicine, Busan 49241, Korea; 3 School of Electronic and Electrical Engineering, Sungkyunkwan University, Suwon 16419, Korea; hyunjinp@skku.edu; 4 Center for Neuroscience Imaging Research, Institute for Basic Science, Suwon 16419, Korea; 5 Department of Health Sciences and Technology, SAIHST, Sungkyunkwan University, Seoul 06355, Korea; 6 Biostatistics and Clinical Epidemiology Center, Samsung Biomedical Research Institute, Seoul 06351, Korea; jhguy.ahn@samsung.com (J.H.A.); insuks@gmail.com (I.S.); 7 Department of Electronic Electrical and Computer Engineering, Sungkyunkwan University, Suwon 16419, Korea; victor87822@gmail.com; 8 Core Research and Development Center, Korean University Ansan Hospital, Ansan 15355, Korea; 9 Department of Thoracic and Cardiovascular Surgery, Samsung Medical Center, Sungkyunkwan University School of Medicine, Seoul 06351, Korea; jkimsmc@skku.edu

**Keywords:** lung neoplasms, adenocarcinoma of lung, prognosis, multi-detector computed tomography, tumor microenvironment

## Abstract

**Simple Summary:**

The tumor microenvironment is a dynamic area, with continuous interaction between tumor cells and their surrounding environment. We aimed to investigate the relationship between tumor radiomic margin characteristics and prognosis in patients with lung cancer. When compared to the model with clinical variables only (C-index = 0.738), the model incorporating clinical variables and radiomic margin characteristics (C-index = 0.753) demonstrated a higher C-index for predicting overall survival. In the model integrating both clinical variables and radiomic margin characteristics, convexity, Laplace of Gaussian (LoG) kurtosis 3, and roundness factor were independent predictive factors of overall survival. Our study showed that radiomic margin characteristics helped predict overall survival in patients with lung adenocarcinomas, thus implying that the tumor margin contains prognostic information.

**Abstract:**

We aimed to investigate the relationship between tumor radiomic margin characteristics and prognosis in patients with lung cancer. We enrolled 334 patients who underwent complete resection for lung adenocarcinoma. A quantitative computed tomography analysis was performed, and 76 radiomic margin characteristics were extracted. The radiomic margin characteristics were correlated with overall survival. The selected clinical variables and radiomic margin characteristics were used to calculate a prognostic model with subsequent internal and external validation. Nearly all of the radiomic margin characteristics showed excellent reproducibility. The least absolute shrinkage and selection operator (LASSO) method was used to select eight radiomic margin characteristics. When compared to the model with clinical variables only (C-index = 0.738), the model incorporating clinical variables and radiomic margin characteristics (C-index = 0.753) demonstrated a higher C-index for predicting overall survival. In the model integrating both clinical variables and radiomic margin characteristics, convexity, a Laplace of Gaussian (LoG) kurtosis of 3, and the roundness factor were each independently predictive of overall survival. In addition, radiomic margin characteristics were also correlated with the micropapillary subtype, and the sphericity value was able to predict the presence of the micropapillary subtype. In conclusion, our study showed that radiomic margin characteristics helped predict overall survival in patients with lung adenocarcinomas, thus implying that the tumor margin contains prognostic information.

## 1. Introduction

In contrast to a distinct barrier between tumor cells and the surrounding parenchyma, the tumor margin is an extremely dynamic area composed of immune cells, rich vasculature, lymphatics, fibroblasts, pericytes, and adipocytes [1,2,3,4]. In other words, the continuous interaction between tumor cells and these non-tumorous cells creates the tumor microenvironment (TME) [1,3,4,5]. Therefore, it has become increasingly clear that the TME plays a critical role in tumor metastasis and prognosis [1,4,6]. In fact, the past decade has demonstrated a paradigm shift in cancer treatment from conventional chemotherapy and radiation therapy to the latest options of angiogenesis-inhibitors and immunotherapy, which focus on controlling the TME instead of directly targeting the tumor cells [7].

Radiomics is a rapidly emerging field that refers to the analysis of large amounts of advanced quantitative features extracted from medical images, and has shown potential in oncology [8,9,10]. In detail, previous studies exploring the radiomics of lung cancer have shown associations with prognosis and treatment response [11,12,13]. However, to our knowledge, few investigators have pursued radiomics features focusing on the TME [14,15]. In this immune-oncology era of lung cancer, the importance of the TME is now greater than ever, and conventional radiomics features using the whole tumor may be missing relevant information located in the environment immediately surrounding the tumor. Therefore, we developed a number of radiomic features that characterized the tumor margin—in other words, radiomic margin characteristics. We anticipated that our developed radiomic margin characteristics would reflect the overall biological process of the TME.

In terms of pathology, many investigators have recognized the importance of the micropapillary (MP) pattern in lung adenocarcinomas. Regardless of the predominant subtype, the presence of the MP subtype is known to be a distinct marker for poor survival in lung adenocarcinomas [16,17,18]. Nitadori et al. reported a higher risk of recurrence in patients with early-stage lung cancers with an MP component of 5% or greater, and suggested that limited resection may be inappropriate for these patients [19]. Furthermore, some researchers have suggested that patients with tumors of the MP pattern may benefit from adjuvant chemotherapy after surgical resection [20,21]. Therefore, pre-operative recognition of the presence of the MP pattern within lung adenocarcinoma may be important for optimal surgical planning, and may advance candidate selection for aggressive post-operative adjuvant therapy.

Therefore, the purpose of this study was two-fold. First, we tested the stability of our radiomic margin characteristics. Next, we investigated whether our radiomic margin characteristics had an association with overall survival and the pathologic MP subtype.

## 2. Materials and Methods

### 2.1. Patients

**Figure 1:**
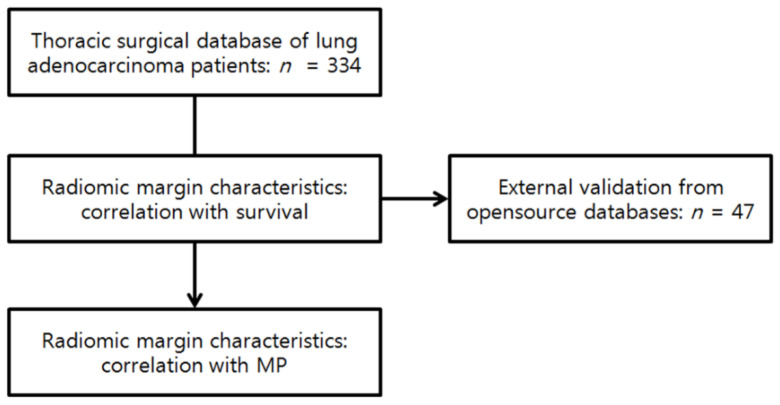
Flow chart of patients, including external validation.

Data from July 2003 to August 2011 in the thoracic surgical database were retrospectively reviewed, and we identified all patients that satisfied the following inclusion criteria: (1) complete surgical resection without neoadjuvant treatment; (2) pre-operative chest computed tomography (CT) scans within 2 weeks before surgery, and with an axial reconstruction interval ≤2.5 mm; (3) comprehensive histologic subtyping, according to the International Association for the Study of Lung Cancer (IASLC)/the American Thoracic Society (ATS)/the European Respiratory Society (ERS) lung adenocarcinoma classification system; and (4) clinical information that could be obtained from the electronic medical records. Among the 339 patients identified, 5 patients were excluded due to indistinct tumor margins with the surrounding parenchyma, due to combined atelectasis or pneumonia. Our final study group included 334 patients (184 men, 150 women; mean age, 60.9 ± 9.96 years; range, 32–86 years) (Figure 1). These patients were part of the cohort from a previous large radiomics study [22].

**Figure 2:**
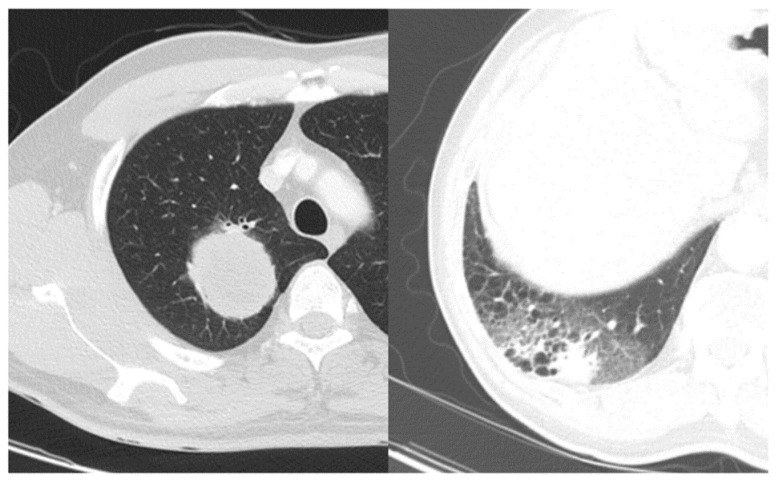
Comparison of lung adenocarcinoma with well-defined smooth margin (**left**) and ill-defined irregular margin (**right**).

External validation for survival was performed using an independent dataset of 47 patients with completely resected lung adenocarcinomas. The imaging parameters of the validation set were comparable to the training set. The CT images of these 47 patients were downloaded from the Cancer Genome Atlas database (https://portal.gdc.cancer.gov/) accessed on 10 August 2017 and the Cancer Imaging Archive database (www.cancerimagingarchive.net/) accessed on 11 August 2017. The radiomic margin characteristics were extracted in the same way as in the original study group for all patients in the external validation group (Figure 2).

### 2.2. Pathology Review

For all patients, the entire tumor specimen was placed on a slide at intervals of 10 mm; thus, a minimum of 3 hematoxylin- and eosin-stained slides were reviewed per patient (range = 3 to 10 slides/patient) by an experienced lung pathologist. According to the 2011 IASLC/ATS/ERS lung adenocarcinoma classification, histologic subtyping was performed semi-quantitatively in 5% increments, and the most predominant pattern was identified. In addition, specifically for the MP pattern, when the MP component constituted ≥1% of the entire tumor, the tumor was subclassified as being positive for the MP subtype [9,16].

### 2.3. Features of Radiomic Margin Characteristics

The CT images were obtained with the following parameters: detector collimation, 1.25 or 0.625 mm; 120 kVp; 150–200 mA; and a reconstruction interval of 1–2.5 mm. For tumor segmentation, using a semi-automated process, two radiologists independently defined the region of interest (ROI) for the whole tumor on the serial axial conventional CT images displayed at a lung window setting [22]. From the ROI, 76 quantitative CT radiomics features characterizing the tumor margin—namely the radiomic margin characteristics—were extracted. Briefly, the features were classified into four categories: (1) shape features; (2) histogram features, after applying Laplace of Gaussian (LoG) filters; (3) 2D shape features; and (4) fractal-based features (fractal dimension, fractal signature dissimilarity (FSD), and lacunarity). The shape features are the most basic, and define the morphology of a tumor. The histogram features represent the range and frequency of the tumor pixel values within the defined lesion ROI. The LoG requires the combination of using a Gaussian smoothing filter to reduce noise in the CT, and then applying a Laplacian filter to highlight regions of rapid intensity change; it is therefore often used for edge detection. In this study, we used multiple scales of 1, 1.5, 2.0, 2.5, 3.0, and 3.5 voxels for the LoG. The 2D shape features are the roundness factor, eccentricity, and solidity. The fractal-based (box-counting) features are based on mathematical measurements that reflect the intrinsic shape of a tumor. The FSD has previously been suggested as a novel image texture analysis technique that uses the blanket method [23]. Detailed mathematical definitions regarding the adopted features are given in Table S1 [8,23,24,25].

### 2.4. Statistical Analysis

The extracted radiomic margin characteristic values were normalized to be between 0 and 1. To test the reliability of extracted radiomic margin characteristics, the intraclass correlation coefficient (ICC) values were calculated by two radiologists.

For the prediction of survival, overall survival (OS) was defined as the time interval between the date of surgical resection and the date of death or last follow-up. The large number of radiomic margin characteristics was reduced using the least absolute shrinkage and selection operator (LASSO) method. A comparison between a stepwise multivariate logistic regression model with clinical features only, and a model integrating both clinical features and selected radiomic margin characteristics, was performed, with the calculation of C-indices included. Next, the internal validation of our prediction model was performed using 10-fold cross-validation. Finally, external validation of our prediction model was performed in a different study group by using the Cox regression, and time-dependent area under the curve (AUC) was calculated. Due to the absence of several clinical variables in the external validation group, which was downloaded from an open data source, external validation was performed with commonly existing clinical variables and all radiomic margin characteristics.

For the prediction of the MP subtype, the large number of radiomic margin characteristics was reduced using the LASSO method. Using radiomics features only, a model predicting the MP pattern was generated using logistic regression and split data validation.

All statistical analyses were performed with Statistical Analysis System (SAS version 9.4; SAS Institute, Cary, NC, USA) and R (version 3.3.1; Vienna, Austria; http://www.R-project.org/) software (accessed on 23 March 2020). *p* < 0.05 indicated statistical significance.

## 3. Results

### 3.1. Stability of Radiomic Margin Characteristics

As shown in Table 1, the ICC values ranged from 0.607 to 1.000. Nearly all radiomic margin characteristics demonstrated excellent reproducibility (ICC values 0.8–1.0), and only two LoG-filtering features demonstrated good reproducibility (ICC values 0.6–0.799). The median 1 and median 1.5 showed ICC values of 0.607 and 0.792, respectively.

### 3.2. Association with Survival

Figure S1 shows the results of the selection of radiomic margin characteristics for the prediction of OS, using the LASSO method. Among 76 radiomic margin characteristics, the eight radiomic margin characteristics of convexity, surface to volume ratio, LoG maximum 3, LoG median 3, LoG minimum 3, LoG kurtosis 3, roundness factor, and FSD blanket were selected. Using these selected radiomic margin characteristics and clinical features, a model predicting survival was generated, with a C-index value of 0.753 (Table 2). For the purpose of comparison, another model using only clinical features to predict survival was generated, with a C-index value of 0.738 (Table 3). The model incorporating both radiomic margin characteristics and clinical features demonstrated better results.

For the internal validation of our survival prediction model using both clinical and radiomic margin characteristics, a ten-fold cross-validation test was performed and good results were obtained for predicting OS (*p* < 0.001).

**Figure 3:**
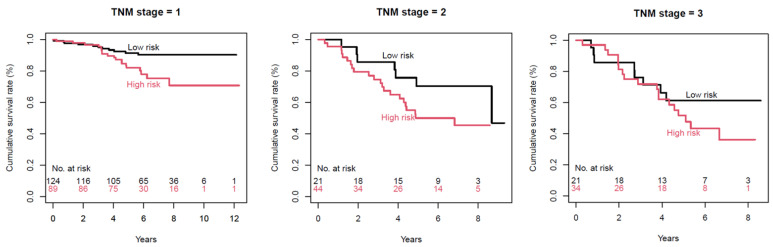
Kaplan–Meier survival curves by TNM stage, using radiomic margin characteristics. *p*-values for low-risk (black) versus high-risk (red) are 0.01, 0.1, and 0.3 for TNM stages 1, 2, and 3, respectively.

Figure 3 depicts Kaplan–Meier survival curves stratified by TNM stage, using radiomic margin characteristics. Only 1 patient had TNM stage 4, and there were 213, 65, and 55 patients at TNM stages 1, 2, and 3, respectively. *p*-values were 0.01, 0.1, and 0.3 for TNM stages 1, 2, and 3, respectively.

**Figure 4:**
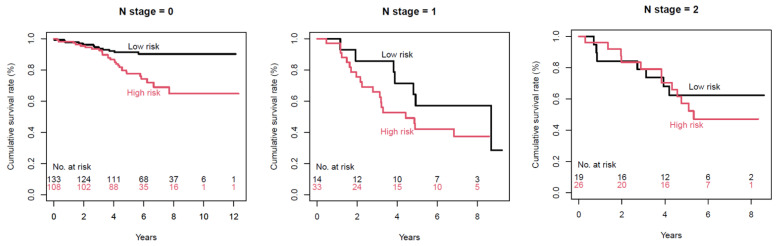
Kaplan–Meier survival curves per lymph node status, using radiomic margin characteristics. *p*-values were 0.0006, 0.2, and 0.6 for N0, N1, and N2, respectively.

Figure 4 displays Kaplan–Meier survival curves stratified by post-operative lymph node status, using radiomic margin characteristics. *p*-values were 0.0006, 0.2, and 0.6 for N0, N1, and N2, respectively.

### 3.3. External Validation of Survival

The external validation study group included 47 patients (28 men and 19 women; the mean age was 68.1 ± 10 years). The mean OS of the external validation group was 10.6 months. Among the 47 patients, 24 (51.1%) patients died (Table S2).

When commonly existing clinical variables in both the training group and the validation group were used, the survival prediction model demonstrated a C-index of 0.747 (*p* = 0.025) (Table S3). Using commonly existing clinical variables and all radiomic margin characteristics, the survival prediction model showed a higher C-index of 0.778 (*p* = 0.024) (Table S4). External validation was performed using these two models. The time-dependent AUC indicated that the model incorporating clinical variables and radiomic margin characteristics performed better at predicting an early survival of less than 20 months (Figure S2).

### 3.4. Association with the MP Pattern

Among our study group of 334 patients, 188 patients (56.3%) did not have the MP pattern, and 146 patients (43.7%) had the MP pattern present. Thirteen patients had the MP-predominant subtype. Figure S3 demonstrates the results of the selection of radiomic margin characteristics for the prediction of the MP pattern using the LASSO method. Among the radiomic margin characteristics, only sphericity value was selected. Using logistic regression and split data validation, only the sphericity value was included in the model for the prediction of the MP pattern, with an AUC of 0.603 (Table 4).

Table S5 shows the percentages of the MP subtype according to the predominant subtype of lung adenocarcinoma. The solid-predominant subtype demonstrated the lowest percentage (6.9%) of the coexisting MP subtype. The acinar- (51.7%) and papillary-predominant (66.7%) subtypes had a higher proportion of the coexisting MP subtype.

## 4. Discussion

The TME is a natural extension of the tumor peripheral area, and includes a variety of tumor-reprogrammed stromal cells [26]. Many investigations have explored the TME to reveal the secrets of tumor progression and discover advanced biomarkers for therapeutic modulation. However, despite the abundant radiomics literature in the field of lung cancer, radiomics investigations of the TME area are relatively scarce [14,15]. We believe that the present study is unique because our radiomic margin characteristics were able to portray the complex TME and reflect the pathologic spatial distribution of the MP subtype in lung adenocarcinomas.

In terms of survival, the C-index values were higher for a model integrating both clinical features and radiomic margin characteristics, compared to those of a model with clinical features only, suggesting that combining clinical and imaging parameters may enhance our knowledge of lung adenocarcinomas. Although the difference may seem small, we acknowledge this as an important small step indicating that radiomics indeed improves our knowledge in the field of lung cancer.

**Figure 5:**
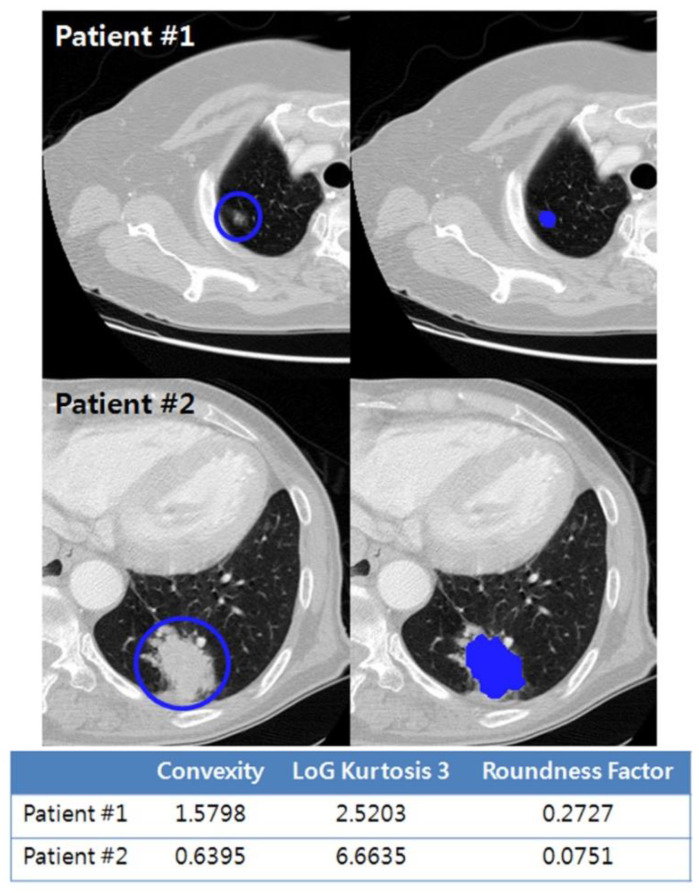
Representative radiomic margin characteristics were extracted from the tumor region of interest and compared between two patients with lung adenocarcinoma. Compared to patient #1, patient #2 demonstrates lower convexity, higher kurtosis, and lower roundness factor values, suggesting worse survival.

In the model integrating both clinical features and radiomic margin characteristics, the three marginal radiomics features, convexity, roundness factor, and LoG kurtosis 3, were independent predictive factors of OS (Figure 5). Convexity quantifies the shape variation in the tumor border; thus, a lower value of convexity represents a more irregular tumor. The roundness factor determines the circularity of a 2D tumor based on its area and perimeter. In the present study, a lower convexity value and a lower roundness factor were associated with worse survival. When compared to a smooth peripheral margin on the CT scan, the spiculated and lobulated margins generally reflect the extension and infiltration of tumor tissues into adjacent lung parenchyma—implying the uneven growth rate in a tumor—and are known to be negative predictors of patient survival [27,28,29]. In current clinical practice, these traditional qualitative (semantic) features of lobulation and spiculation have shown significant associations with clinical endpoints, including OS; yet, intra-observer and inter-observer variability remains a major problem of semantic features [27,30,31]. According to Yip et al., the binary or categorical scales employed to rate sematic features may be insufficient to describe subtle tumor characteristics. In contrast, radiomics features have values that are on a continuous scale, which can provide greater detail for changes in tumor characteristics [15]. Therefore, the novelty of our study is that we succeeded in the objective quantification of the tumor margin characteristics through our developed radiomic margin characteristics, and we correlated them with OS.

In addition, higher kurtosis after applying the LoG spatial filter with coarse texture (scale of 3.0) was associated with worse survival in the present study. Kurtosis, the peakedness of the distribution of pixel values, is increased by intensity variations in the highlighted objects [32]. Many factors in the TME may lead to increased kurtosis, including our speculation of increased micro-vessels and a large amount of immune cells in the TME. It is well-known that angiogenesis is fundamental to tumor growth, and increases the opportunity for cancer cells to enter the bloodstream, along with increasing the chance of metastasis [33]. Meert et al. conducted a meta-analysis, and reported that pathologic micro-vessel count reflected angiogenesis, and was a poor prognostic factor for survival in patients with lung cancer [34]. Similarly, Parra et al. reported that tumor-associated inflammatory cells were more prominent in the peritumoral region compared to the intratumoral area, and were associated with prognosis [35].

Interestingly, when stratified according to the TNM stages, a trend of better survival discrimination was observed in early-stage patients, and radiomic margin characteristics showed the best clinical utility in TNM stage 1 patients. Furthermore, when stratified by post-operative lymph node status, radiomic margin characteristics showed the best performance in N0 patients. These results suggest that radiomics may help in determining patients with higher risks—even in early-stage lung adenocarcinomas.

Histologically, the MP pattern is defined as tumor cells growing in papillary structures, with tufts lacking a central fibrovascular core and floating in alveolar spaces, thus attributing to a more invasive and metastatic behavior [36]. Miyoshi et al. reported that the MP pattern demonstrates disruption of the cell-to-cell adhesion complex, which can act as a basis for its invasive spread [37]. In addition, the spread through air spaces, which refers to the MP cells spreading within air spaces beyond the edge of the main tumor, has also been recognized as a pattern of invasion in lung cancer [38,39,40]. Thus, we correlated our radiomic margin characteristics with the MP subtype, and by using the LASSO method, the only radiomic margin characteristic selected was sphericity. Using logistic regression and split data validation, only the sphericity value remained in the model. The sphericity value represents the overall spherical shape of a tumor, and a less spherical shape was associated with the presence of the MP subtype in the present study. Although there are few studies, the MP subtype has been reported to be more commonly seen scattered at the periphery, rather than at the center of the tumor [6,41]. Our radiomic margin characteristic of sphericity may reflect this spatial distribution of the MP subtype.

Last, an important hurdle in developing the clinical application of radiomics features is reproducibility. In our study, with regard to ICC values, we found that our developed radiomic margin characteristics demonstrated excellent reproducibility. The description of radiomics analysis differs substantially among published studies, and any identification of reliable or relevant quantitative information for tumor characterization must be reproducible. 

Our study had several limitations. First, the external validation group was rather small, and was downloaded from open-access databases, including medical images collected from all over the world. Thus, the CT image datasets are extremely heterogeneous, and these variabilities may have weakened the statistical power of our external validation group. In addition, some clinical variables and whole-tumor histologic subtyping data were not available from the open-access database; thus, we could not perform external validation for the prediction of the MP subtype, and only performed external validation using commonly existing clinical variables for OS. Furthermore, there was a considerable survival difference between the training group and the validation set. To be specific, among the 334 patients of the training group, there were only 10 deaths during the first year. In contrast, during the same period of one year, there were 17 deaths among the 47 patients in the validation set. Second, five patients were excluded, as the tumor margins were obscured due to combined atelectasis and obstructing pneumonia surrounding the tumor. Accurate tumor margin delineation is mandatory for the extraction of radiomic margin characteristics reflecting the TME. Therefore, future investigations regarding tumor boundary segmentation methods may enhance the accuracy and quality of the extracted radiomic margin characteristics. Third, variations in CT slice reconstruction in the training set may have affected radiomic analysis. We tried to minimize this by excluding patients with CT images reconstructed at larger than 2.5 mm slice thickness. Fourth, although we provided speculations about the significant radiomics features, the exact biological, pathological interpretation is rather limited and beyond the scope of this study. Fifth, we did not compare our radiomics features with subjective scores rated by radiologists. Lastly, deep learning methods can extract relevant imaging features aside from the current approach of radiomics. Due to the limited samples in the medical imaging field, we could fine-tune pre-trained convolutional neural network models derived from natural or medical images, and use the flattened intermediate feature maps as the imaging features. We plan to adopt such approaches in the future.

## 5. Conclusions

In conclusion, our developed radiomic margin characteristics showed good reproducibility, and were correlated with OS and the MP subtype. We suggest that this study provides a radiomics basis for understanding the TME, and that it could potentially be used as an imaging-based prognostic biomarker in lung adenocarcinomas to promote related research.

## Figures and Tables

**Table 1:** Distribution of intraclass correlation coefficient (ICC) values according to the 4 categories. Nearly all radiomic margin characteristics showed excellent reproducibility.

Category	Shape	LoG Filtering	2D Shape	Fractal-Based
Total number of features in each category	7	63	3	3
Range of ICC	0.904–0.994	0.607–1.000	0.868–0.958	0.935–0.955
Number of features with ICC ≥0.8	7 (100%)	61 (96.8%)	3 (100%)	3 (100%)
Number of features with ICC 0.6–0.799	0 (0%)	2 (3.2%)	0 (0%)	0 (0%)

**Table 2:** Model integrating clinical features and radiomic margin characteristics for the prediction of overall survival (C-index: 0.753).

Selected Variables	Reference	*p*-Value	OR	95% CI
MP (Predominant cell non-solid)		0.022	1.959	1.100–3.490
MP (Predominant cell is solid)		0.010	2.539	1.253–5.148
Moderately differentiated	Well-differentiated	0.023	3.946	1.208–12.890
Poorly differentiated	Well-differentiated	0.036	4.110	1.098–15.389
Sex	Male	0.035	1.669	1.036–2.688
Age		<0.001	1.047	1.021–1.073
Size		0.019	1.219	1.034–1.438
Convexity		0.004	0.078	0.013–0.447
LoG Kurtosis 3		0.034	1.085	1.006–1.170
Roundness factor		0.009	2.384	1.246–4.561

Note—OR, odds ratio; CI, confidence interval; MP, micropapillary pattern.

**Table 3:** Model with clinical variables only for the prediction of overall survival (C-index: 0.738).

Selected Variables	Reference	*p*-Value	OR	95% CI
MP (Predominant cell non-solid)		0.018	1.985	1.122–3.512
MP (Predominant cell is solid)		0.006	2.730	1.335–5.583
Moderately differentiated	Well-differentiated	0.045	3.317	1.026–10.731
Poorly differentiated	Well-differentiated	0.074	3.299	0.891–12.214
Sex	Male	0.033	1.677	1.043–2.698
Age		<0.001	1.048	1.023–1.073
Size		0.004	1.228	1.066–1.414

Note—OR, odds ratio; CI, confidence interval; MP, micropapillary pattern.

**Table 4:** The result of logistic regression and split data validation for predicting the micropapillary subtype (area under the curve (AUC): 0.603, 97% CI: 0.489–0.716).

Variable	*p*-Value	OR	95% CI
(Intercept)	0.059		
Sphericity value	0.033	0.071	0.006–0.813

Note—OR, odds ratio; CI, confidence interval.

## Data Availability

The data presented in this study are available on request from the corresponding author. The data are not publicly available due to local Institutional Review Board regulation.

## Supplementary Materials

The following are available online at https://www.mdpi.com/article/10.3390/cancers13071676/s1, Figure S1: Selection of radiomics features for the prediction of overall survival using the LASSO logistic regression model, Figure S2: (A) Survival curves of the validation group stratified by the median of predicted survival according to the model incorporating both clinical variables and radiomics features; (B) Time-dependent AUC demonstrates that the model incorporating clinical variables and radiomics features showed better performance for predicting early survival of less than 20 months, Figure S3: Selection of radiomics features for the prediction of the MP subtype using the LASSO logistic regression model, Table S1: Definition of extracted radiomic features, Table S2: Patient characteristics of the thoracic surgical database and external validation group, Table S3: Model only using commonly existing clinical variables for the prediction of overall survival (C-index: 0.747) for external validation, Table S4: Model using commonly existing clinical variables and all radiomics features for the prediction of overall survival (C-index: 0.778) for external validation, Table S5: Percentages of MP subtype according to the predominant subtype of lung adenocarcinoma.
