# Supplementary material for: Tumor Margin Contains Prognostic Information: Radiomic Margin Characteristics Analysis in Lung Adenocarcinoma Patients
Source: Cancers (Basel). 2021 Apr 2;13(7):1676. doi: 10.3390/cancers13071676 (PMC8037340; doi:10.3390/cancers13071676)
Supplement: Supplementary file 1 [file cancers-13-01676-s001.pdf]

*Supplementary Materials*

# Tumor Margin Contains Prognostic Information: Radiomic Margin Characteristics Analysis in Lung Adenocarcinoma Patients

**Geewon Lee, Hyunjin Park, Ho Yun Lee, Joong Hyun Ahn, Insuk Sohn, Seung-Hak Lee and Jhingook Kim**

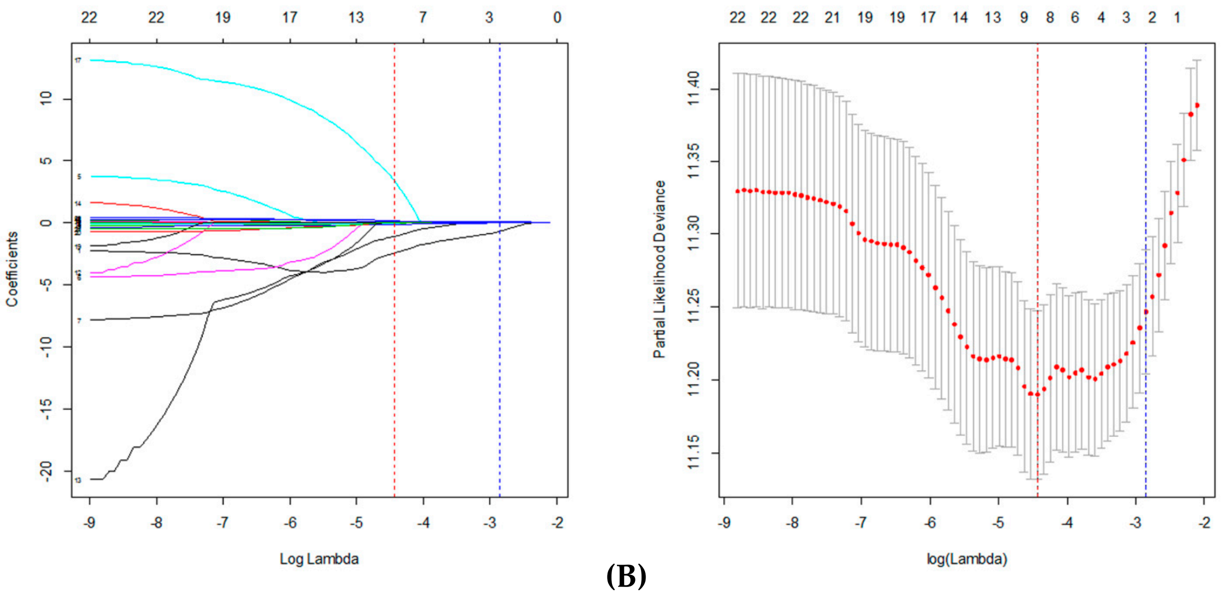


**(A)** **(B)**

**Figure S1.** Selection of radiomics features for the prediction of overall survival using the LASSO logistic regression model. (**A**) LASSO coefficients produced by the regression analysis. Eight marginal radiomics features were selected. (**B**) Selection of the tuning parameter (lambda) in the LASSO model via LOOCV (leave one out cross-validation). Dotted red and blue vertical lines were drawn at the optimal values by minimum criteria and 1-s.e. criteria, respectively.

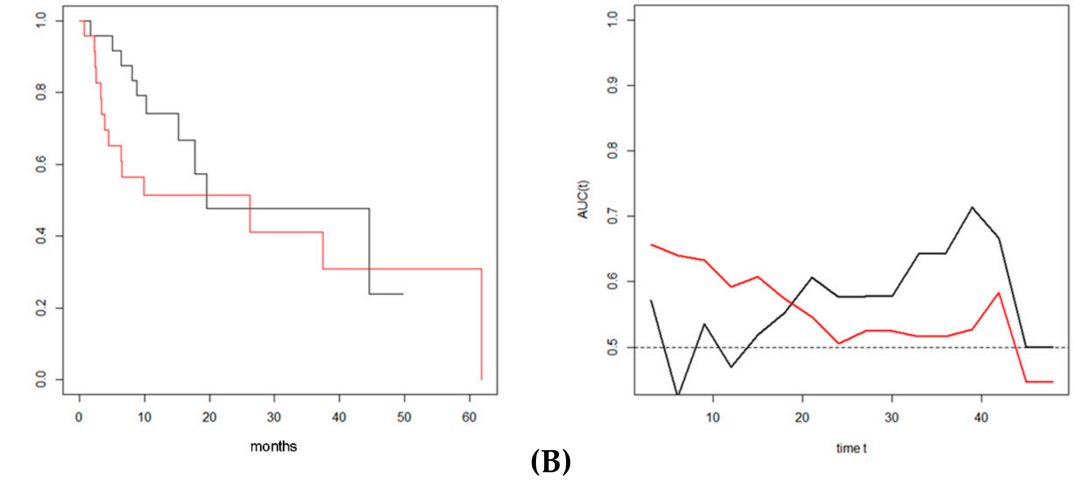


**(A)** **(B)**

**Figure S2.** (**A**) Survival curves of the validation group stratified by the median of predicted survival according to the model incorporating both clinical variables and radiomics features. (Red line, group with lower survival; Black line, group with better survival). (**B**) Time-dependent AUC demonstrates that the model incorporating clinical variables and radiomics features showed better performance for predicting early survival of less than 20 months (Red line, clinical variables and radiomics features; Black line, clinical variables only).

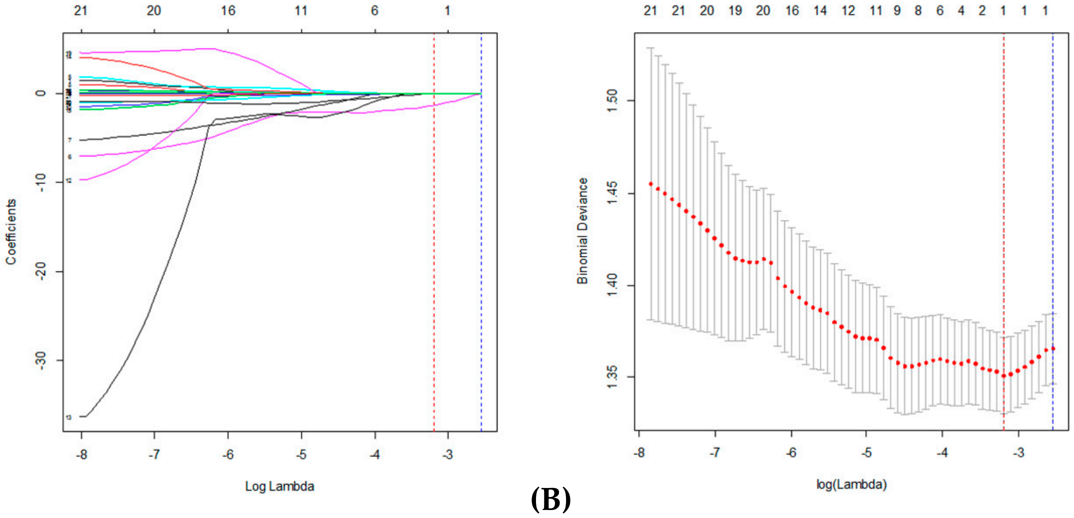


**(A)** **(B)**

**Figure S3.** Selection of radiomics features for the prediction of the MP subtype using the LASSO logistic regression model. (**A**) LASSO map shows coefficients plotted against the log(lambda) sequence. Only the sphericity value radiomics feature (pink line, 6th variable) was selected. (**B**) LASSO coefficient analysis of the radiomics features. Using LOOCV (leave one out cross-validation), the minimum value of log(lambda) was found to be −1.373. Dotted red and blue vertical lines were drawn at the optimal values by minimum criteria and 1-s.e. criteria, respectively.

**Table S1.** Definition of extracted radiomics features.

| | Parameter | Formula | Description |
|---|---|---|---|
| Filter-based features (LoG) [19] | Mean | $\text{Mean} = \frac{1}{N}\sum_{i}^{N} G(i)$ Where G denote the filtered 3d image matrix with N voxel. | Measurement of mean of ROI image processed by LoG filter |
| | Max | $\text{Max} = Max(G(i))$ Where G denotes the filtered 3d image matrix with $N$ voxel. | Measurement of max intensity value of ROI image processed by LoG filter |
| | Min | $\text{Min} = Min(G(i))$ Where G denotes the filtered 3d image matrix with $N$ voxel. | Measurement of minimum intensity value of ROI image processed by LoG filter |
| | Median | $\text{Median} = \frac{G(i)}{2}$ Where G denote the filtered 3d image matrix | Measurement of median intensity value of ROI image processed by LoG filter |
| | Standard deviation (Std) | $\text{Std} = \left(\frac{1}{N-1}\sum_{i=1}^{N} (G(i) - \bar{G})^2\right)^{1/2}$ Where G denote the filtered 3d image matrix with N voxel. | Measurement of standard deviation of ROI image processed by LoG filter |
| | Skewness | $\text{Skewness} = \frac{E(G-\mu)^3}{\sigma^3}$ Where $\mu$ is the mean of G, $\sigma$ is the standard deviation of G, $E$ is the expectation operator. | Measurement of skewness of ROI image processed by LoG filter |
| | Kurtosis | $\text{Kurtosis} = \frac{E(G-\mu)^4}{\sigma^4}$ Where $\mu$ is the mean of G $\sigma$ is the standard deviation of G, $E$ is the expectation operator. | Measurement of kurtosis of ROI image processed by LoG filter |
| | Uniformity | $\text{Uniformity} = \sum_{i=1}^{N_l} P(i)^2$ Where $P$ denotes the first-order histogram with $N_l$ discrete intensity levels. | Measurement of uniformity of ROI image processed by LoG filter |
| Shape- and size-based features [8,19] | Compactness | $\text{Compactness} = \frac{V}{\sqrt{\pi}A^{\frac{3}{2}}}$ Where $V$ denotes the volume, and $A$ denotes the surface area of the volume of interest (VOI) | Quantifies how close an object is to the smoothest shape, the circle |
| | Surface area | $\text{SA} = \sum_{i=1}^{N}\frac{1}{2}\lvert a_i b_i \times a_i c_i\rvert$ Where $N$ is the total number of triangles (coved surface area), and $a, b, c$ are edge vectors | The surface area of the ROI |
| | Convexity | $\text{Convexity} = \frac{V}{V'}$ Where $V$ denotes tumor volume, and $V'$ denotes convex hull volume | Measures ratio of the ROI volume contained within the tumor to the calculated convex hull volume |
| | Sphericity | $\text{Sphericity} = \frac{\pi^{\frac{1}{3}} \times (6V)^{\frac{2}{3}}}{A}$ Where $A$ denotes area, and $V$ denotes tumor volume | Measures of the roundness of the ROI |
| | Spherical disproportion | $\text{Spherical disproportion} = \frac{A}{4\pi R^2}$ Where $R$ is the radius of a sphere with the same volume as the tumor | The ratio of the surface area of the ROI to the surface area of a sphere with the same volume as the ROI |
| | Maximum 3D diameter | See description in the next column | Measures of the maximum 3D ROI diameter. It is measured as the largest pairwise Euclidean distance, between surface voxels of the ROI |
| | Surface to volume ratio (SVR) | $\text{SVR} = \frac{A}{V}$ Where $A$ is area, and $V$ is volume | Surface to volume ratio in ROI |
| | Volume | $\text{Volume} = R * \text{number of voxels}$ Where R denote the 3d image resolution | Volume of tumor (ROI) |
| | Mass | $\text{Mass} = V * D$ Where V denote the tumor volume, D denotes the tumor density | Mass of tumor (ROI) |
| | Density | $\text{Density} = \frac{M}{V}$ | Density of tumor (ROI) |

| | | | |
|---|---|---|---|
| | | Where $V$ denote the tumor volume, $M$ denote the tumor mass | |
| | Roundness factor (2D) | $\text{Roundness factor} = \frac{4\pi \cdot Area}{Perimeter^2}$ | Measure of circularity of a ROI |
| | Eccentricity (2D) | Eccentricity = c/a<br>Where c is the distance from the center to a focus and a is the distance from that focus to a vertex | Measure of how the tumor shape is close to the circle |
| | Solidity (2D) | $\text{Solidity} = \frac{Area}{Convex\ area}$ | Measure of convexity of a ROI on the 2D image |
| Fractal-based features [18,20] | Lacunarity (Box-counting method) | See description in the next column | Measure of the texture or distribution of gaps within an image |
| | Dimension (Box-counting method) | $\text{Fractal dimension} = \lim_{r \to 0} \frac{\log(N_r)}{\log(1/r)}$<br>Where $N_r$ is the number of voxels, and $r$ is the each of different side lengths | Fractal dimension quantifies morphological complexity and provides information on the self-similarity properties |
| | Fractal signature dissimilarity (Blanket method) | See description in the next column | Measure of tumor heterogeneity infromation |

Note–LoG, Laplacian of Gaussian; ROI, region of interest.

**Table S2.** Patient characteristics of the thoracic surgical database and external validation group.

| | **Thoracic Surgical Database** ***N*** **= 334** | **External Validation** ***N*** **= 47** |
|---|---|---|
| Sex | | |
| Male | 184 | 28 |
| Female | 150 | 19 |
| Age (Mean ± SD) | 60.9 ± 9.96 | 68.1 ± 10 |
| Overall Survival (Mean) | 66.7 months | 10.6 months |
| Death | 85 (25.4%) | 24 (51.1%) |

**Table S3.** Model only using commonly existing clinical variables for the prediction of overall survival (C-index: 0.747) for external validation.

| Selected Variables | Reference | *p* Value | OR | 95% CI |
|---|---|---|---|---|
| Sex | Male | 0.012 | 1.820 | 1.142–2.903 |
| Age | | <0.001 | 1.042 | 1.018–1.067 |
| TNM stage 2 | TNM stage 1 | <0.001 | 3.422 | 2.040–5.740 |
| TNM stage 3 | TNM stage 1 | <0.001 | 4.247 | 2.496–7.227 |

Note–OR, odd's ratio; CI, confidence interval.

**Table S4.** Model using commonly existing clinical variables and all radiomics features for the prediction of overall survival (C-index: 0.778) for external validation.

| Selected Variables | Reference | *p* Value | OR | 95% CI |
|---|---|---|---|---|
| Sex | Male | 0.007 | 1.926 | 1.195–3.104 |
| Age | | <0.001 | 1.593 | 1.244–2.041 |
| TNM stage 2 | TNM stage 1 | <0.001 | 2.823 | 1.589–5.016 |
| TNM stage 3 | TNM stage 1 | <0.001 | 4.505 | 2.558–7.933 |
| Convexity | | <0.001 | 0.567 | 0.413–0.779 |
| LoG Uniformity 0.5 | | 0.042 | 0.693 | 0.486–0.987 |
| LoG Kurtosis 1 | | <0.001 | 1.100 | 1.044–1.159 |
| Roundness Factor | | <0.001 | 4.257 | 2.094–8.653 |
| Lacunarity | | 0.021 | 0.570 | 0.353–0.920 |

Note–OR, odd's ratio; CI, confidence interval.

**Table S5.** Percentages of MP subtype according to the predominant subtype of lung adenocarcinoma.

| Predominant Subtype (%) | Lepidic | Acinar | Papillary | MP | Solid |
|---|---|---|---|---|---|
| MP absent | 68.8 | 48.3 | 33.3 | 0 | 93.2 |
| MP present | 31.3 | 51.7 | 66.7 | 100 | 6.9 |

Note: MP, micropapillary; * Due to rounding, not all percentages total 100.

© 2021 by the authors. Licensee MDPI, Basel, Switzerland. This article is an open access article distributed under the terms and conditions of the Creative Commons Attribution (CC BY) license (http://creativecommons.org/licenses/by/4.0/).
